# Social Dilemmas in Nature-Based Tourism Depend on Social Value Orientations

**DOI:** 10.1038/s41598-020-60349-z

**Published:** 2020-02-28

**Authors:** Keita Honjo, Takahiro Kubo

**Affiliations:** 1 Global Warming Countermeasures Group, Center for Environmental Science in Saitama (CESS), Kamitanadare 914, Kazo, Saitama Prefecture, 347-0115 Japan; 2 Center for Environmental Biology and Ecosystem Studies, National Institute for Environmental Studies (NIES), Onogawa 16-2, Tsukuba, Ibaraki Prefecture, 305-8506 Japan

**Keywords:** Sustainability, Applied mathematics

## Abstract

Nature-based tourism (NBT) is vulnerable to a rapid increase in visitors because natural resources are often open access. Market failure caused by over-exploitation of natural resources is an example of social dilemmas in common-pool resource systems. Game theory, which describes people’s decision making under conflicts, has been applied to the analysis of social dilemmas in NBT. However, previous studies use non-cooperative games assuming individualistic players and discuss the emergence of social dilemmas only in a limited situation. Here, we demonstrate, by developing a two-player non-cooperative game of wildlife viewing, that the traditional game-theoretic approach fails to find social dilemmas. By analysing the competition between tour operators (players) with different social value orientations (SVOs), we found that concentration of tours becomes a Pareto-inefficient Nash equilibrium (PINE) when both players are competitive. Whether the wildlife-viewing market is a Prisoner’s dilemma depends on players’ SVOs. Furthermore, we found that fair punishment on competitive players promotes rather than suppresses the emergence of PINE. Our results suggest that the diversity of SVOs is an essential factor in understanding social dilemmas in NBT.

## Introduction

Tourism demand has been rapidly increasing in recent years. According to the World Travel and Tourism Council^1^, travel and tourism contributed USD 8,272 billion (at 2017 constant prices and exchange rates) to world GDP in 2017. The amount is expected to reach USD 12,450 billion in 2028. Meanwhile, many tourism destinations face a rapid increase in visitors with the associated problems: degraded tourist experiences, damage to nature, and threats to culture and heritage^2^. Nature-based tourism (NBT) is vulnerable to overcrowding because natural resources are often open access. On an analogy with the tragedy of the commons^3,4^, we can imagine the tourism dilemma as follows: (1) Self-interested tourism firms increase tour supply to maximise their benefits. (2) Over-exploitation of natural resources happens, undermining the basis of NBT. (3) The economic value of NBT decreases, and the payoff allocation to tourism firms becomes inefficient. The tourism dilemma is an example of social dilemmas in common-pool resource systems^5–7^.

Game theory, which describes people’s decision making under conflicts, is a useful tool to analyse social dilemmas in common-pool resource systems^8^. There are many game-theoretic studies in the fields of fisheries, water resource management, and climate change^9–11^. Game theory is not widely applied to NBT, but several studies discuss tourism dilemmas based on non-cooperative games. For example, Sobhee and co-workers^12^ use the Prisoner’s dilemma to represent the conflict between fishers and water-sport operators in a marine park. Blanco and co-workers^13^ propose a strategic-form game structurally different from the Prisoner’s dilemma, pointing out that many tourism firms voluntarily engage in resource conservation. In another paper^14^, they demonstrate, in an evolutionary extension of the above game, that sustainable and non-sustainable firms can coexist in a sufficiently large market. He and co-workers^15^ also use an evolutionary game to investigate how government supervision affects the behaviour of tourism firms and visitors. Bimonte^16^ represents the conflict between residents and visitors using a Bayesian game and concludes that incomplete information causes social dilemmas. Pirotta and Lusseau^17^ develop an agent-based model of wildlife viewing and analyse the interactions of tour operators, visitors, and animals. By numerical simulation, they evaluate the impacts of resource conservation policies (e.g. taxation, subsidies, and cap-and-trade mechanisms) on the profits of tour operators.

Game-theoretic studies on NBT aim to evaluate the effects of resource conservation policies by developing mathematical models of tourism dilemmas. However, most previous studies discuss tourism dilemmas in limited situations, assuming individualistic players. The individualistic player maximises his or her own but does not consider others’ payoffs. Since the publication of *Theory of Games and Economic Behavior* in 1944^18^, the individualistic player assumption has been widely applied to non-cooperative games. On the other hand, psychological experiments demonstrated that individuals have social preferences and make decisions by comparing the payoffs for themselves and others^19–27^. To examine the relationships between personality traits and human behaviour, we need to consider utility functions which include information about opponents’ payoffs. A generalised model of social preferences is social value orientation (SVO)^22,25,26^. Mathematically, SVO is expressed as a function that calculates each person’s utility from the payoffs for self and others. The SVO model can describe the behaviour of players with different personality traits (e.g. individualistic, competitive, prosocial, altruistic, and sadistic). Despite the achievements of psychological studies, the diversity of personality traits gathers little attention in game-theoretic studies on tourism dilemmas.

This paper focuses on the tourism dilemma in wildlife viewing^28–31^, which is a popular form of NBT. We represent the competition between tour operators by a two-player non-cooperative game and identify mathematical conditions under which the market falls into social dilemmas. In other words, we identify the combinations of parameters that produce Pareto-inefficient Nash equilibria (PINE) as the solutions in the Wildlife-Viewing game (WVG). Furthermore, we impose a punishment on tour concentration and examine its impact on the emergence of PINE. To overcome the limitation of previous studies, we assume three types of players with different SVOs (individualistic, competitive, and prosocial) and solve the WVG for all SVO combinations. The concept of our game originates with wildlife-viewing tours conducted in the Amami Oshima of Japan. One of the target animals is the Amami rabbit (*Pentalagus furnessi*), which is an endangered endemic species^32,33^. The concentration of tours hinders the activities of the Amami rabbit and harms the environment suitable for rabbit viewing. According to Kubo and co-workers^31^, tour operators in the Amami Oshima use money-back guarantee systems to attract visitors. If a visitor fails to view the Amami rabbit in a tour, the tour operator returns a part of the tour fee to the visitor. The WVG includes parameters corresponding to these characteristics.

This paper is constructed as follows. In the Methods section, we give the mathematical expressions of the WVG and PINE. In the Results section, we calculate the Nash equilibria, Pareto-efficient solutions, and PINE of the game. Moreover, we calculate the solutions of the WVG with punishment for tour concentration. In the Discussion section, we summarise the results and conclude the paper by pointing out the limitations of our approach. Supplementary Information provides details of mathematical calculation.

## Methods

### Informal description of the WVG

Before introducing a mathematical expression of the WVG, we provide an informal description of the game. The WVG represents the competition between two operators who are running tours for viewing an endemic rabbit. An area inhabited by the rabbit is open to people, and no one has the right to own the area. Anyone can visit the viewing area by paying a cost. However, information about the viewing area is not open to visitors, and they cannot encounter the rabbit without an operator’s guidance. The operators supply wildlife-viewing tours to visitors and receive fees from them. This paper considers wildlife viewing as a common-pool resource consumed by individuals. The rabbit is sensitive to human-generated noise and leaves the viewing area if two operators conduct tours simultaneously. If an operator fails to encounter the rabbit during a tour, he or she returns a part of the tour fee to the visitor (money-back guarantee). As each operator’s net profit can be negative, he or she needs to consider the other operator’s action in conducting a tour. In the WVG, the operators make decisions based on utility rather than monetary payoffs. Each operator evaluates the utility of action by comparing monetary payoffs for the self and the other. The operator’s personality trait (SVO) influences the evaluation of utility. For simplicity, we do not consider the process in which the operators gather visitors. Moreover, we assume that the tour fee, cost, and money-back rate are constant regardless of the operators. Under these conditions, we examine whether the wildlife-viewing market falls into social dilemmas.

### Mathematical expression of the WVG

Consider a situation where two players operate tours for viewing an endemic rabbit. Let **N** = {1, 2} be the set of players. Each player can conduct a tour by paying a cost *C* > 0. If a player encounters the rabbit during a tour, it receives a tour fee *B* > 0 from the visitor. If a player fails to find the rabbit, it returns a part of the tour fee to the visitor and receives *δB*. *δ* ∈ (0, 1) is called the guarantee coefficient. Each player encounters the rabbit with a probability *P* ∈ [0, 1]. However, if the two players conduct tours simultaneously, the noise they generate threatens the rabbit, and the encounter probability decreases to *θP*. *θ* ∈ (0, 1) is called the vigilance coefficient. Let **S** = {T, F} be the set of pure strategies. A player with strategy T conducts a tour, while a player with strategy F does not. When each player *i* ∈ **N** chooses strategy *s*_*i*_ ∈ **S**, his or her expected payoff is given by

1 $${U}_{i}^{{s}_{1}{s}_{2}}=\left\{\begin{array}{ll}{\theta }^{m-1}PB+(1-{\theta }^{m-1}P)\delta B-C & ({s}_{i}={\rm{T}})\\ 0 & ({s}_{i}={\rm{F}})\end{array}\right.,$$

where *m* ∈ {1, 2} is the number of players with strategy T. Using equation (1), we define the objective payoff bimatrix (OPBM) as follows:

2 $$U=\left[\begin{array}{ll}({U}_{1}^{{\rm{TT}}},{U}_{2}^{{\rm{TT}}}) & ({U}_{1}^{{\rm{TF}}},{U}_{2}^{{\rm{TF}}})\\ ({U}_{1}^{{\rm{FT}}},{U}_{2}^{{\rm{FT}}}) & ({U}_{1}^{{\rm{FF}}},{U}_{2}^{{\rm{FF}}})\end{array}\right]=\left[\begin{array}{ll}({U}_{1}^{{\rm{TT}}},{U}_{1}^{{\rm{TT}}}) & ({U}_{1}^{{\rm{TF}}},0)\\ (0,{U}_{1}^{{\rm{TF}}}) & (0,0)\end{array}\right].$$

Each player determines his or her strategy based on the subjective payoff bimatrix (SPBM) rather than the OPBM. Psychological studies propose mathematical functions for payoff transformation^19–27^. We use the most fundamental function, which calculates each player’s subjective payoff as a linear combination of the objective payoffs for the self and the other:

3 $${V}_{i}^{{s}_{1}{s}_{2}}={\alpha }_{i}{U}_{i}^{{s}_{1}{s}_{2}}+{\beta }_{i}{U}_{-i}^{{s}_{1}{s}_{2}}\quad (-i\in {\bf{N}},i\ne -\,i).$$

**Figure 1:**
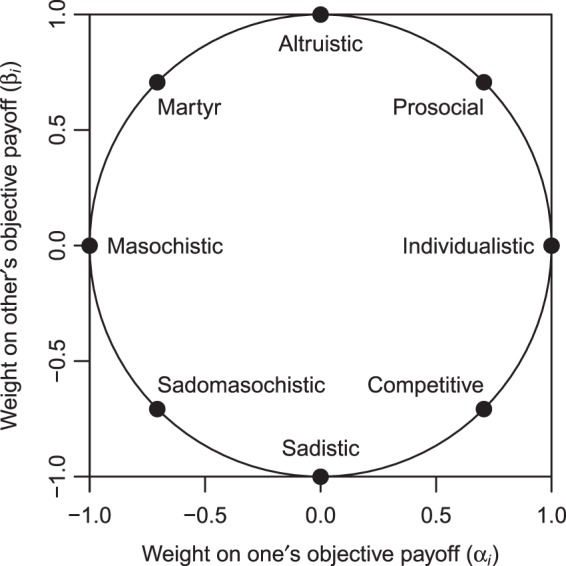
Social value orientation (SVO) ring^26^.

*α*_*i*_ ∈ [− 1, 1] and *β*_*i*_ ∈ [−1, 1] are the weights on the objective payoffs for the self and the other, respectively. To normalise the subjective payoff vector, we assume that ${\alpha }_{i}^{2}+{\beta }_{i}^{2}=1$. A weight vector (*α*_*i*_, *β*_*i*_) indicates player *i*’s SVO. The set of all weight vectors draws the SVO ring on the two-dimensional space ${{\mathbb{R}}}^{2}$ (Fig. 1). We focus on three typical SVOs with positive values of *α*_*i*_: individualistic, competitive, and prosocial. The individualistic player, who is characterised by the weight vector (1, 0), maximises his or her own but does not consider the other’s objective payoff. Non-cooperative games usually assume individualistic players. The competitive and prosocial players are characterised by the weight vectors $(1/\sqrt{2},-1/\sqrt{2})$ and $(1/\sqrt{2},1/\sqrt{2})$, respectively. With an increase in the other’s objective payoff, the subjective payoff decreases for the competitive player but increases for the prosocial player. From equations (2) and (3), we obtain the SPBM:

4 $$V=\left[\begin{array}{ll}({V}_{1}^{{\rm{TT}}},{V}_{2}^{{\rm{TT}}}) & ({V}_{1}^{{\rm{TF}}},{V}_{2}^{{\rm{TF}}})\\ ({V}_{1}^{{\rm{FT}}},{V}_{2}^{{\rm{FT}}}) & ({V}_{1}^{{\rm{FF}}},{V}_{2}^{{\rm{FF}}})\end{array}\right]=\left[\begin{array}{ll}(({\alpha }_{1}+{\beta }_{1}){U}_{1}^{{\rm{TT}}},({\alpha }_{2}+{\beta }_{2}){U}_{1}^{{\rm{TT}}}) & ({\alpha }_{1}{U}_{1}^{{\rm{TF}}},{\beta }_{2}{U}_{1}^{{\rm{TF}}})\\ ({\beta }_{1}{U}_{1}^{{\rm{TF}}},{\alpha }_{2}{U}_{1}^{{\rm{TF}}}) & (0,0)\end{array}\right].$$

### Mathematical expression of PINE

Here, we show the mathematical expression of PINE. The Nash equilibrium is the set of strategy profiles where every player shows the best response to the other’s strategy^34,35^. In the WVG, players make decisions based on the SPBM. Hence, the Nash equilibrium is

5 $${{\bf{S}}}_{{\rm{Nash}}}^{2}=\{({s}_{1}^{\ast },{s}_{2}^{\ast })| \forall i\in {\bf{N}}(\forall {s}_{i}\in {\bf{S}}({V}_{i}({s}_{i}^{\ast },{s}_{-i}^{\ast })\ge {V}_{i}({s}_{i},{s}_{-i}^{\ast })))\},$$

where *V*_*i*_(*s*_*i*_, *s*_−*i*_) denotes the subjective payoff for player *i*.

**Figure 2:**
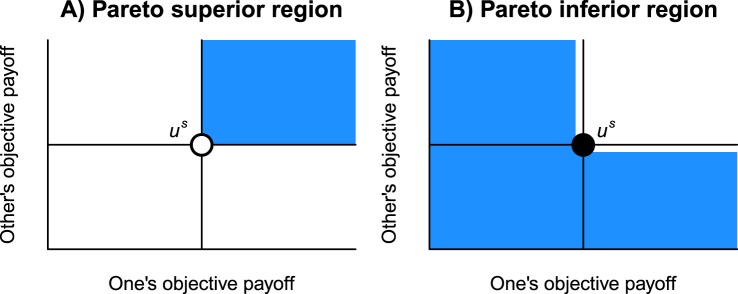
Pareto superior and inferior regions with respect to an objective payoff allocation *u*^*s*^.

An objective payoff allocation is Pareto efficient if no player can increase his or her objective payoff without decreasing the other’s^35,36^. Let ${u}^{s}=({U}_{1}^{s},{U}_{2}^{s})$ be the objective payoff allocation given by a strategy profile *s* ∈ **S**^2^. We define the region Pareto superior to *u*^*s*^ as ${\bf{P}}({u}^{s})=[{U}_{1}^{s},\infty )\times [{U}_{2}^{s},\infty )\backslash \{{u}^{s}\}$ (Fig. 2A). By moving from *u*^*s*^ to any point of **P**(*u*^*s*^), at least one player can improve his or her objective payoff without decreasing the other’s. The complement of the Pareto-superior region, denoted by ${{\mathbb{R}}}^{2}\backslash {\bf{P}}({u}^{s})$, is the Pareto-inferior region (Fig. 2B). A strategy profile *σ* ∈ **S**^2^ is a Pareto-efficient solution if all possible objective payoff allocations are Pareto inferior to *u*^*σ*^. The set of Pareto-efficient solutions is

6 $${{\bf{S}}}_{{\rm{Pareto}}}^{2}=\{\sigma | \forall s\in {{\bf{S}}}^{2}({u}^{s}\in {{\mathbb{R}}}^{2}\backslash {\bf{P}}({u}^{\sigma }))\}.$$

From equations (5) and (6), we obtain the mathematical expression of the PINE:

7 $${{\bf{S}}}_{{\rm{Nash}}}^{2}\wedge ({{\bf{S}}}^{2}\backslash {{\bf{S}}}_{{\rm{Pareto}}}^{2}).$$

The WVG is a social dilemma if it has a PINE as the solution.

## Results

### Distribution of nash equilibria

**Figure 3:**
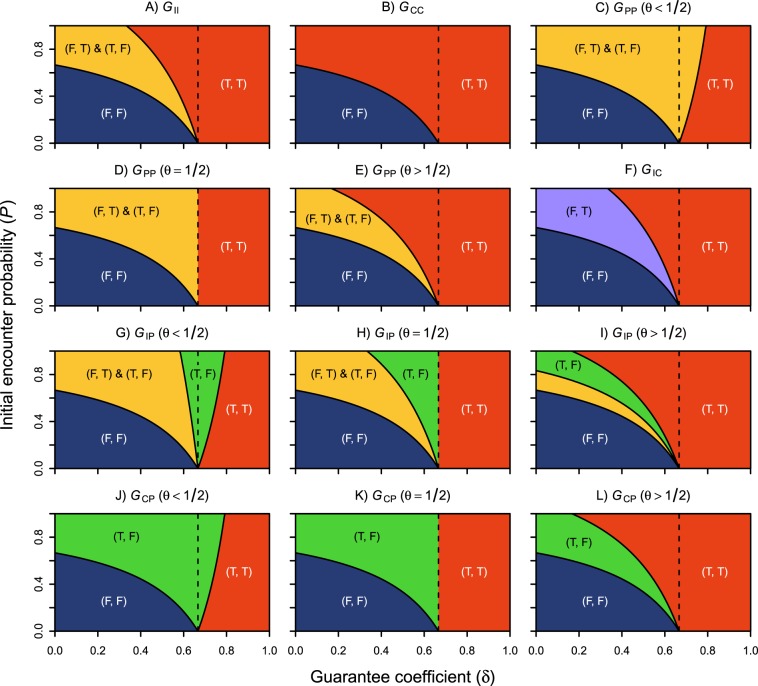
Nash equilibria of the subjective payoff bimatrix (SPBM) for different combinations of parameters (*B* = 1.5, *C* = 1.0, and *θ* ∈ {0.2, 0.5, 0.8}). Panels C, G, and J assume *θ* = 0.2. Panels E, I, and L assume *θ* = 0.8. The other panels assume *θ* = 0.5. The solution conditions are listed in Tables S1–S3 of Supplementary Information. ${G}_{{\tau }_{1}{\tau }_{2}}$ denotes a WVG with player *i* of type *τ*_*i*_ ∈ {I, C, P}. The symbols I, C, and P are abbreviations for individualistic, competitive, and prosocial types, respectively. The dashed line indicates *δ* = *C*/*B*.

First, we identified the conditions under which the SPBM (equation (4)) has Nash equilibria. Section 1 of Supplementary Information describes the calculation process in detail, and Tables S1–S3 list the equilibrium conditions. Let **D** = {(*δ*, *P*) ∣ *δ* ∈ (0, 1), *P* ∈ [0, 1]} be a two-dimensional space spanned by the guarantee coefficient and the initial encounter probability. Figure 3 shows the distribution of Nash equilibria over the parameter space **D**. ${G}_{{\tau }_{1}{\tau }_{2}}$ denotes a WVG with player *i* of type *τ*_*i*_ ∈ {I, C, P}. The symbols I, C, and P are abbreviations for individualistic, competitive, and prosocial types, respectively. The dashed line indicates *δ* = *C*/*B*. The result is summarised in the following two theorems:

#### Theorem 1.

*Consider a two-player WVG. Each player is individualistic, competitive, or prosocial. The SPBM of the game always has at least one pure-strategy Nash equilibrium*.

*Proof*. The conditions for Nash equilibria are shown in Tables S1–S3 of Supplementary Information. The union of the conditions for (F, F)-Nash, (F, T)-Nash, (T, F)-Nash, and (T, T)-Nash is *P* ∈ [0, 1] regardless of players’ SVOs. Hence, the SPBM always has at least one of the Nash equilibria.

#### Theorem 2.

*Assume that the guarantee coefficient* (*δ*) *and the initial encounter probability (P) are randomly determined. The probability of tour concentration (i.e. the probability of* (T, T)-*Nash) in the competitive market* (*G*_CC_) *is equal to or higher than that in the individualistic market* (*G*_II_). *In contrast, the probability of tour concentration in the prosocial market* (*G*_PP_) *is equal to or lower than that in the individualistic market*.

*Proof*. Let ${{\bf{D}}}_{{\rm{Nash}}}^{{\rm{TT}}}({G}_{{\tau }_{1}{\tau }_{2}})\subseteq {\bf{D}}$ be a parameter region where the SPBM of a game ${G}_{{\tau }_{1}{\tau }_{2}}$ has (T, T)-Nash. When a parameter vector (*δ*, *P*) is randomly chosen from **D**, the area of the parameter region, denoted by $| {{\bf{D}}}_{{\rm{Nash}}}^{{\rm{TT}}}({G}_{{\tau }_{1}{\tau }_{2}})|$, gives the probability of (T, T)-Nash. By definition, $| \varnothing | =0$. Consider the case of *δ* < *C*/*B*. From Table S1, we obtain ${{\bf{D}}}_{{\rm{Nash}}}^{{\rm{TT}}}({G}_{{\rm{II}}})=\{(\delta ,P)| \delta \in (0,C/B),P\in [{P}_{0}/\theta ,1]\}$ and ${{\bf{D}}}_{{\rm{Nash}}}^{{\rm{TT}}}({G}_{{\rm{CC}}})=\{(\delta ,P)| \delta \in (0,C/B),P\in [{P}_{0},1]\}$, where *P*_0_ = (*C* − *δB*)/(*B*(1 − *δ*)). ${{\bf{D}}}_{{\rm{Nash}}}^{{\rm{TT}}}({G}_{{\rm{PP}}})$ is {(*δ*, *P*) ∣ *δ* ∈ (0, *C*/*B*), *P* ∈ [*P*_0_/(2*θ* − 1), 1]} under *θ* > 1/2 and is the empty set under *θ* ≤ 1/2. We have $[{P}_{0},1]\supseteq [{P}_{0}/\theta ,1]\supseteq \varnothing$ for any *θ* ∈ (0, 1). Moreover, we have [*P*_0_, 1] ⊇ [*P*_0_/*θ*, 1] ⊇ [*P*_0_/(2*θ* − 1), 1] when *θ* > 1/2. Hence, we obtain ${{\bf{D}}}_{{\rm{Nash}}}^{{\rm{TT}}}({G}_{{\rm{CC}}})\supseteq {{\bf{D}}}_{{\rm{Nash}}}^{{\rm{TT}}}({G}_{{\rm{II}}})\supseteq {{\bf{D}}}_{{\rm{Nash}}}^{{\rm{TT}}}({G}_{{\rm{PP}}})$, which results in $| {{\bf{D}}}_{{\rm{Nash}}}^{{\rm{TT}}}({G}_{{\rm{CC}}})| \ \ge \ | {{\bf{D}}}_{{\rm{Nash}}}^{{\rm{TT}}}({G}_{{\rm{II}}})| \ge | {{\bf{D}}}_{{\rm{Nash}}}^{{\rm{TT}}}({G}_{{\rm{PP}}})|$. This inequality also holds in the case of *δ* ≥ *C*/*B* (Tables S2 and S3). Thus, Theorem 2 is proved.

Here, we focus on the emergence of (T, T)-Nash, which implies concentration of tours. When the guarantee coefficient is lower than *C*/*B* (the left side of the dashed line), the failure of a tour gives a negative profit (*δB* − *C* < 0). In this case, the distribution of Nash equilibria changes depending on players’ SVOs. The game *G*_II_ yields the solution (T, T)-Nash when *P* ∈ [*P*_0_/*θ*, 1]. In the game *G*_CC_, (T, T)-Nash emerges when *P* ∈ [*P*_0_, 1]. Since *θ* ∈ (0, 1), the parameter region of (T, T)-Nash is broader in *G*_CC_ than in *G*_II_. The competitive player aggressively conducts a tour even with low initial encounter probability. Therefore, tour concentration frequently occurs in the competitive market. In the game *G*_PP_, (T, T)-Nash emerges only in limited conditions: *θ* > 1/2 and *P* ∈ [*P*_0_/(2*θ* − 1), 1]. Since *P*_0_/*θ* < *P*_0_/(2*θ* − 1) under the constraint of *θ* > 1/2, the parameter region of (T, T)-Nash is narrower in *G*_PP_ than in *G*_II_. Unlike the competitive player, the prosocial player avoids tour concentration even with high initial encounter probability. However, the prosocial player is vulnerable to competition with the individual and competitive players. The games *G*_IP_ and *G*_CP_ can have (T, F)-Nash as the solutions. Under this equilibrium, the prosocial player stops the supply of tours, and the individualistic or competitive player monopolises the market. Similarly, the individualistic player is also vulnerable to competition with the competitive player. The game *G*_IC_ can lead to the solution (F, T)-Nash. When the guarantee coefficient is higher than or equal to *C*/*B* (the right side of the dashed line), a tour failure results in a non-negative profit (*δB* − *C* ≥ 0). In this case, (T, T)-Nash is dominant in most of the games.

### Distribution of pareto-efficient solutions

**Figure 4:**
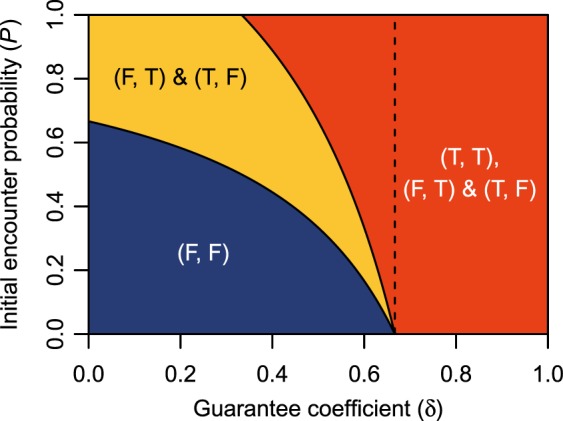
Pareto-efficient solutions of the objective payoff bimatrix (OPBM) for different combinations of parameters (*B* = 1.5, *C* = 1.0, and *θ* = 0.5). Table S4 of Supplementary Information lists the solution conditions. The dashed line indicates *δ* = *C*/*B*.

Second, we identified the conditions under which the OPBM (equation (2)) has Pareto-efficient solutions. Section 2 of Supplementary Information describes the calculation process in detail, and Table S4 lists the solution conditions. As the individual, competitive, and prosocial players have the same OPBM, the solution conditions are independent of players’ SVOs. Figure 4 shows the distribution of Pareto-efficient solutions over the parameter space **D**. We have the following theorem:

#### Theorem 3.

*The OPBM of the WVG always has at least one Pareto-efficient solution*.

*Proof*. The conditions for Pareto-efficient solutions are shown in Table S4 of Supplementary Information. The union of the conditions for (F, F)-Pareto, (F, T)-Pareto, (T, F)-Pareto, and (T, T)-Pareto is *P* ∈ [0, 1]. Hence, the OPBM always has at least one of the Pareto-efficient solutions.

### Conditions for PINE

Finally, we identified the conditions under which the WVG has PINE as the solutions. Tables S5 and S6 of Supplementary Information list the solution conditions. The result is summarised in the following theorem:

#### Theorem 4.

*Assume that the guarantee coefficient* (*δ*) *and the initial encounter probability* (*P*) *are randomly determined. (1) Consider the case where at least one player is individualistic or prosocial. The objective payoff allocation given by a Nash equilibrium of the SPBM is almost surely Pareto efficient. Non-competitive markets fall into social dilemmas with probability zero. (2) Consider the case where both players are competitive. If the guarantee coefficient is lower thanC*/*Band the initial encounter probability lies in the interval* [*P*_0_, *P*_0_/*θ*], *the objective payoff allocation given by a Nash equilibrium of the SPBM is not Pareto efficient. If* *B* > *C*, *the competitive market falls into a social dilemma with a positive probability*.

*Proof*. Let ${{\bf{D}}}_{{\rm{PINE}}}({G}_{{\tau }_{1}{\tau }_{2}})\subseteq {\bf{D}}$ be a parameter region where a game ${G}_{{\tau }_{1}{\tau }_{2}}$ has at least one PINE, and let $| {{\bf{D}}}_{{\rm{PINE}}}({G}_{{\tau }_{1}{\tau }_{2}})|$ be the area of the parameter region. When a parameter vector (*δ*, *P*) is randomly chosen from **D**, $| {{\bf{D}}}_{{\rm{PINE}}}({G}_{{\tau }_{1}{\tau }_{2}})|$ gives the probability of PINE. By definition, $| \varnothing | =0$. The conditions for PINE are shown in Tables S5 and S6. The union of the conditions for (F, F)-PINE, (F, T)-PINE, (T, F)-PINE, and (T, T)-PINE is non-empty only in the games *G*_II_, *G*_CC_, and *G*_IC_ with *δ* < *C*/*B*. From Table S5, we obtain ∣**D**_PINE_(*G*_II_)∣ = ∣{(*δ*, *P*)∣*δ* ∈ (0, *C*/*B*), *P* = *P*_0_/*θ*}∣ = 0, ∣**D**_PINE_(*G*_CC_)∣ = ∣{(*δ*, *P*)∣*δ* ∈ (0, *C*/*B*), *P* ∈ [*P*_0_, *P*_0_/*θ*]}∣≥0, and ∣**D**_PINE_(*G*_IC_)∣ = ∣**D**_PINE_(*G*_II_)∣ = 0. Under the constraint of *B* > *C*, we have *P*_0_ < 1, which results in ∣**D**_PINE_(*G*_CC_)∣ > 0. Thus, Theorem 2 is proved.□

**Figure 5:**
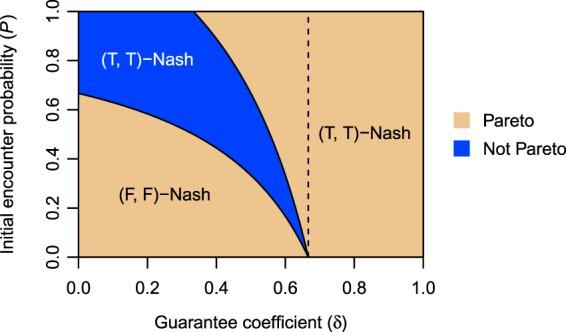
Pareto efficiency in the objective payoff allocations given by Nash equilibria of the game *G*_CC_ (*B* = 1.5, *C* = 1.0, and *θ* = 0.5). Tables S5,S6 of Supplementary Information list the solution conditions. In the blue region, the WVG has PINE. The dashed line indicates *δ* = *C*/*B*.

Figure 5 shows the distribution of PINE in the game *G*_CC_. In the blue region, the Nash equilibrium is (T, T), but the Pareto-efficient solutions are (F, T) and (T, F). Here, we provide a numerical example of PINE. Consider the case where *B* = 1.5, *C* = 1.0, *δ* = 0.3, *P* = 0.6, and *θ* = 0.6. By equations (2) and (4), the OPBM and SPBM are respectively

8 $$U=\left[\begin{array}{ll}(-0.17,-0.17) & (0.08,0)\\ (0,0.08) & (0,0)\end{array}\right]$$

and

9 $$V=\left[\begin{array}{ll}(0,0) & (0.06,-0.06)\\ (-0.06,0.06) & (0,0)\end{array}\right].$$

In this case, T is the dominant strategy for both players, and the SPBM has the unique Nash equilibrium (T, T). The equilibrium gives the objective payoff allocation (− 0.17, − 0.17), which is not Pareto efficient. This objective payoff allocation becomes a Pareto-efficient solution (0, 0.08) or (0.08, 0) if one player changes his or her strategy. However, no player changes his or her strategy because the shift from strategy T to strategy F decreases the player’s subjective payoff from 0 to −0.06.

### Failure of fair punishment in the competitive market

We demonstrated that the strategy profile (T, T), which implies tour concentration, becomes a PINE in the game *G*_CC_. Competitive players cannot escape from PINE without an incentive to change their strategies. Here, we impose punishment on the objective payoff allocation from (T, T) and examine its impact on the emergence of PINE.

We define the OPBM with punishment as

10 $${U}_{\epsilon }=\left[\begin{array}{ll}({U}_{1}^{{\rm{TT}}}-\epsilon ,{U}_{1}^{{\rm{TT}}}-\epsilon ) & ({U}_{1}^{{\rm{TF}}},0)\\ (0,{U}_{1}^{{\rm{TF}}}) & (0,0)\end{array}\right],$$

where *ε* > 0 is a penalty on tour concentration. The SPBM is

11 $${V}_{\epsilon }=\left[\begin{array}{ll}(({\alpha }_{1}+{\beta }_{1})({U}_{1}^{{\rm{TT}}}-\epsilon ),({\alpha }_{2}+{\beta }_{2})({U}_{1}^{{\rm{TT}}}-\epsilon )) & ({\alpha }_{1}{U}_{1}^{{\rm{TF}}},{\beta }_{2}{U}_{1}^{{\rm{TF}}})\\ ({\beta }_{1}{U}_{1}^{{\rm{TF}}},{\alpha }_{2}{U}_{1}^{{\rm{TF}}}) & (0,0)\end{array}\right].$$

This punishment is fair in the sense that two players with strategy T are forced to pay an equal penalty. From equation (11), we found that the punishment decreases the parameter region of (T, T)-Nash in all the games except *G*_CC_ (Section 4 of Supplementary Information). In the game *G*_II_, for example, the border between (T, T)-Nash and other equilibria shifts from *P* = *P*_0_/*θ* to *P* = (*P*_0_ + *ε*_0_)/*θ* (Fig. S1A). Since *ε*_0_ = *ε*/(*B*(1 − *δ*)) > 0 and *θ* ∈ (0, 1), the new border is located on the right side of the original border. In the game *G*_PP_ with *θ* > 1/2, similarly, the border between (T, T)-Nash and other equilibria shifts from *P* = *P*_0_/(2*θ* − 1) to *P* = (*P*_0_ + 2*ε*_0_)/(2*θ* − 1) (Fig. S1E). As shown in Fig. S1B, however, the punishment does not change the distribution of Nash equilibria in the game *G*_CC_. By equation (11), the SPBM of *G*_CC_ is

12 $${V}_{\epsilon }=\left[\begin{array}{ll}(0,0) & ({U}_{1}^{{\rm{TF}}}/\sqrt{2},-{U}_{1}^{{\rm{TF}}}/\sqrt{2})\\ (-{U}_{1}^{{\rm{TF}}}/\sqrt{2},{U}_{1}^{{\rm{TF}}}/\sqrt{2}) & (0,0)\end{array}\right]=V.$$

**Figure 6:**
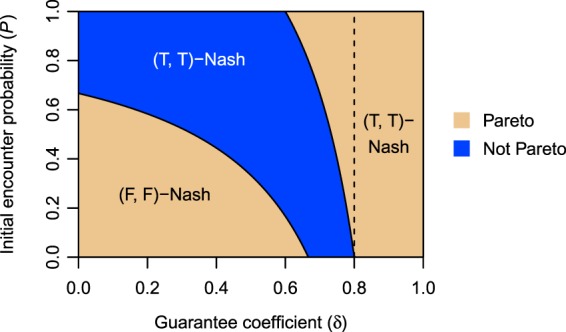
Distribution of PINE in the game *G*_CC_ with fair punishment on the strategy profile (T, T) (*B* = 1.5, *C* = 1.0, *θ* = 0.5, and *ε* = 0.2). In the blue region, the WVG has PINE. The dashed line indicates *δ* = (*C* + *ε*)/*B*.

Thus, the SPBM with punishment is equal to the SPBM without punishment. On the other hand, the distribution of Pareto-efficient solutions is influenced by the punishment (Section 5 of Supplementary Information). The border between (T, T)-Pareto and other solutions shifts from *P* = *P*_0_/*θ* to *P* = (*P*_0_ + *ε*_0_)/*θ*. The parameter region of (T, T)-Pareto under punishment is narrower than the original region (Fig. S2). As a result, the parameter region of PINE in the game *G*_CC_ changes from Fig. 5 to Fig. 6. The result is summarised in the following theorem:

#### Theorem 5.

*Consider a case where punishment on the strategy profile* (T, T) *reduces each player’s objective payoff byϵ* > 0. *When both players are competitive, the parameter region of* (T, T)-*PINE over the parameter space* **D** *is equal to or broader than that in the original game without punishment. If the guarantee coefficient* (*δ*) *and the initial encounter probability* (*P*) *are randomly determined, the punishment monotonically increases the probability that the competitive market falls into a social dilemma*.

*Proof*. Let $${G}_{{\rm{CC}}}^{\varepsilon }$$ be the competitive market with the punishment. The game $${G}_{{\rm{CC}}}^{\varepsilon }$$ has (T, T)-Nash under *P* ∈ [*P*_0_, 1] (Section 4.2 of Supplementary Information) and has (T, T)-Pareto under *P* ∈ ((*P*_0_ + *ε*_0_)/*θ*, 1] (Section 5.2 of Supplementary Information). As the inequality *P*_0_ < (*P*_0_ + *ε*_0_)/*θ* holds over **D**, the condition for (T, T)-PINE is *P* ∈ [*P*_0_, (*P*_0_ + *ε*_0_)/*θ*]. The parameter region of (T, T)-PINE is expressed as $${{\bf{D}}}_{{\rm{PINE}}}^{{\rm{TT}}}({G}_{{\rm{CC}}}^{\varepsilon })=\{(\delta ,P)| \delta \in (0,1),P\in $$$$[{P}_{0},({P}_{0}+{\varepsilon }_{0})/\theta ]\}$$. Since (*P*_0_ + *ε*_0_)/*θ* > *P*_0_/*θ*, we have [*P*_0_, (*P*_0_ + *ε*_0_)/*θ*] ⊇ [*P*_0_, *P*_0_/*θ*]. Hence, we obtain $${{\bf{D}}}_{{\rm{PINE}}}^{{\rm{TT}}}({G}_{{\rm{CC}}}^{\varepsilon })\supseteq {{\bf{D}}}_{{\rm{PINE}}}^{{\rm{TT}}}({G}_{{\rm{CC}}})$$, which results in $$| {{\bf{D}}}_{{\rm{PINE}}}^{{\rm{TT}}}({G}_{{\rm{CC}}}^{\varepsilon })| \ge | {{\bf{D}}}_{{\rm{PINE}}}^{{\rm{TT}}}({G}_{{\rm{CC}}})| $$. When a parameter vector (*δ*, *P*) is randomly chosen from **D**, the areas of the parameter regions give the probabilities of (T, T)-PINE in the games $${G}_{{\rm{CC}}}^{\varepsilon }$$ and *G*_CC_. Thus, Theorem 5 is proved.□

Here, we provide a numerical example of the failure of fair punishment. Assume that *B* = 1.5, *C* = 1.0, *δ* = 0.5, *P* = 0.8, *θ* = 0.6, and *ϵ* = 0.8. The OPBM without punishment is

13 $$U=\left[\begin{array}{ll}(0.11,0.11) & (0.35,0)\\ (0,0.35) & (0,0)\end{array}\right],$$

and the OPBM with punishment is

14 $${U}_{\epsilon }=\left[\begin{array}{ll}(-0.69,-0.69) & (0.35,0)\\ (0,0.35) & (0,0)\end{array}\right].$$

First, we consider the individualistic market (*G*_II_). In this case, the SPBM is equal to the OPBM (*V* = *U* and *V*_*ε*_ = *U*_*ε*_). In the original game, strategy T is the best response for both players, and the SPBM has the unique Nash equilibrium (T, T). The objective payoff allocation (0.11, 0.11) given by the equilibrium is Pareto efficient. With the introduction of fair punishment into the OPBM, the Nash equilibrium shifts to (T, F) or (F, T), and the concentration of tours is suppressed. The objective payoff allocation (0.35, 0) or (0, 0.35) given by the new equilibrium is also Pareto efficient. The total objective payoff increases from 0.22 to 0.35. Second, we consider the competitive market (*G*_CC_). In this case, the SPBM is

15 $$V={V}_{\epsilon }=\left[\begin{array}{ll}(0,0) & (0.25,-0.25)\\ (-0.25,0.25) & (0,0)\end{array}\right],$$

which has the unique Nash equilibrium (T, T). This equilibrium is not influenced by the punishment, but the objective payoff allocation under the punishment, (−0.69, −0.69), is no longer Pareto efficient. The total objective payoff decreases from 0.22 to −1.38.

## Discussion

In this paper, we developed a non-cooperative game of wildlife viewing and demonstrated that the SVOs of tour operators (players) are associated with the emergence of PINE. The WVG describes the competition between individualistic, competitive, and prosocial players in the market. Compared to the individualistic player, the competitive player aggressively conducts a tour even if the probability of encountering the target animal is low (Theorem 2). In contrast, the prosocial player avoids the concentration of tours, which causes a decrease in the tour profit (Theorem 2). The WVG always has at least one pure-strategy Nash equilibrium and has at least one Pareto-efficient solution (Theorems 1 and 3). From the distributions of Nash equilibria and Pareto-efficient solutions over the parameter space, we found that PINE emerge when both players are competitive (Theorem 4). In the competitive market, the concentration of tours becomes a Nash equilibrium, but the objective payoff allocation given by the solution is not always Pareto efficient. If at least one player is individualistic or prosocial, the objective payoff allocation given by the Nash equilibrium is almost surely Pareto efficient. Whether the wildlife-viewing market is a Prisoner’s dilemma depends on players’ SVOs.

To remove PINE from the competitive market, we need to provide players with an incentive to stop tour supply when the encounter probability is low. We imposed fair punishment on tour concentration and examined its impact on the emergence of PINE. If at least one player is individualistic or competitive, the punishment decreases the parameter region where tour concentration is a Nash equilibrium. However, the punishment loses its effect in the competitive market and does not change the distribution of Nash equilibria. Unlike the individualistic player, the competitive player makes decisions based on the difference between the objective payoffs for self and the other. As fair punishment maintains the objective-payoff difference, it does not influence the behaviour of competitive players. On the other hand, the punishment decreases the parameter region where tour concentration is a Pareto-efficient solution. From these results, we immediately find that the punishment increases the parameter region of PINE when both players are competitive (Theorem 5). In other words, fair punishment promotes rather than suppresses the emergence of PINE in the competitive market. Our results suggest the possibility that the traditional game-theoretic approach assuming individualistic players fails to find social dilemmas in NBT.

By assuming players with different SVOs, we demonstrated that the WVG is a potential social dilemma. Although this paper was inspired by the rabbit-viewing tour conducted in the Amami Oshima of Japan, the WVG can represent wildlife-viewing tours not only in Japan but also around the world (e.g. bear, seal, and whale^28–30^). Furthermore, the SVO-based approach adopted in the WVG is widely applicable to various non-cooperative games. We provide three examples in Section 6 of Supplementary Information: the Hawk-Dove game, Battle of the Sexes, and Public Goods game^35,37^. In each of the games, individualistic, competitive, and prosocial players show different behaviours even though they have the same OPBM. Like the WVG, the Hawk-Dove game and Battle of the Sexes are potential social dilemmas in which only competitive players are attracted to PINE. In the Public Goods game, the total objective payoff is maximised when both players are prosocial. The SVO-based approach enables us to find hidden theoretical solutions of non-cooperative games.

We conclude this paper by discussing two limitations of our approach. First, our results do not fully reflect the diversity of personality traits. As illustrated by the SVO ring (Fig. 1), there exist an infinite number of SVOs. This paper focused on the three typical SVOs, but altruism and sadism are also important SVOs associated with social dilemmas^38–43^. Section 7 of Supplementary Information summarises the behaviour of altruistic and sadistic players in the WVG. Unlike the typical SVOs, strategy choices of altruistic and sadistic players are independent of the game parameters. When both players are altruistic or sadistic, the WVG can be a social dilemma. The SVO model used in this paper is the most basic and does not cover the whole set of personality traits. To represent inequity aversion, for example, we need to extend the subjective payoff function using information about the objective-payoff difference^21,24,44^. Psychological studies have developed various personality-trait models (e.g. Big Five, HEXACO, Dark Triad, and Dark Tetrad^40,42,44–50^), which are likely to contribute to further research on the tourism dilemma. Some studies report that equity and efficiency preferences do not always explain people’s decision making. Capraro and co-workers^51,52^ highlight the importance of normative motives (e.g. morality and the belief that God is watching us) for altruistic behaviour in social dilemmas.

Second, the WVG developed in this paper is a single-shot game with two players and assumes a highly simplified tourism market. We can consider various extensions of the WVG. For example, an evolutionary extension of the game enables us to analyse the dynamics of players’ behaviour^37^. If the relationships between players form a social network, local interactions of players influence the state of the whole market^37,53^. Results from evolutionary game theory and psychological experiments suggest that reputation and voluntary punishment promote altruistic behaviour in repeated games on social networks^38,54–58^. As shown by this paper, tour concentration can be a PINE in the competitive market, and the solution is resistant to fair punishment introduced by an external force (e.g. the government). Unfortunately, we could not find a way to remove the PINE from the competitive market. To overcome this problem, we need to analyse the dynamic interactions of players in an extended WVG.

## Supplementary information

Supplementary Information

## Data Availability

The authors declare that the paper and Supplementary Information include all data which are necessary to obtain the results.

## References

[1] World Travel and Tourism Council. Travel and Tourism: Economic Impact 2018 World (2018).

[2] World Travel and Tourism Council. Coping with Success: Managing Overcrowding in Tourism Destinations (2017).

[3] Hardin G (1968). The tragedy of the commons. Sci..

[4] Ostrom, E. *Governing the Commons: The Evolution of Institutions for Collective Action* (Cambridge University Press, 1990).

[5] Healy RG (1994). The common pool problem in tourism landscapes. Annals Tour. Res..

[6] Briassoulis H (2002). Sustainable tourism and the question of the commons. Annals Tour. Res..

[7] Moore SA, Rodger K (2010). Wildlife tourism as a common pool resource issue: Enabling conditions for sustainability governance. J. Sustain. Tour..

[8] Redpath SM (2018). Games as tools to address conservation conflicts. Trends Ecol. Evol..

[9] Bailey M, Sumaila UR, Lindroos M (2010). Application of game theory to fisheries over three decades. Fish. Res..

[10] Madani K (2010). Game theory and water resources. J. Hydrol.

[11] Wood PJ (2011). Climate change and game theory. Annals New York Acad. Sci..

[12] Sobhee SK, Ramessur R, Bhukuth A (2017). A game theory approach to fishers’ strategic behavior vis-à-vis hotel-based water sports operators: The case of the Balaclava Marine Park project in Mauritius. Ocean. Coast. Manag..

[13] Blanco E, Rey-Maquieira J, Lozano J (2009). Economic incentives for tourism firms to undertake voluntary environmental management. Tour. Manag..

[14] Blanco E, Lozano J, Rey-Maquieira J (2009). A dynamic approach to voluntary environmental contributions in tourism. Ecol. Econ..

[15] He P, He Y, Xu F (2018). Evolutionary analysis of sustainable tourism. Annals Tour. Res..

[16] Bimonte S (2008). The tragedy of tourism resources as the outcome of a strategic game: A new analytical framework. Ecol. Econ..

[17] Pirotta E, Lusseau D (2015). Managing the wildlife tourism commons. Ecol. Appl..

[18] von Neumann, J. & Morgenstern, O. *Thoery of Games and Economic Behavior* (Princeton University Press, 1944).

[19] Loewenstein GF, Thompson L, Bazerman MH (1989). Social utility and decision making in interpersonal contexts. J. Pers. Soc. Psychol..

[20] Levine DK (1998). Modeling altruism and spitefulness in experiments. Rev. Econ. Dyn..

[21] Fehr E, Schmidt KM (1999). A theory of fairness, competition, and cooperation. The Q. J. Econ..

[22] Van Lange PAM (1999). The pursuit of joint outcomes and equality in outcomes: An integrated model of social value orientation. J. Pers. Soc. Psychol..

[23] Bolton GE, Ockenfels A (2000). ERC: A theory of equity, reciprocity, and competition. The Am. Econ. Rev..

[24] Charness G, Rabin M (2002). Understanding social preferences with simple tests. The Q. J. Econ..

[25] Bogaert S, Boone C, Declerck C (2008). Social value orientation and cooperation in social dilemmas: A review and conceptual model. Br. J. Soc. Psychol..

[26] Murphy RO, Ackermann KA (2014). Social value orientation: Theoretical and measurement issues in the study of social preferences. Pers. Soc. Psychol. Rev..

[27] Barcelo H, Capraro V (2015). Group size effect on cooperation in one-shot social dilemmas. Sci. Reports.

[28] Kubo T, Shoji Y (2016). Demand for bear viewing hikes: Implications for balancing visitor satisfaction with safety in protected areas. J. Outdoor Recreat. Tour..

[29] Granquist SM, Nilsson P-A (2016). Who’s watching whom? An interdisciplinary approach to the study of seal-watching tourism in Iceland. J. Clean. Prod..

[30] Higham JES, Bejder L, Allen SJ, Corkeron PJ, Lusseau D (2016). Managing whale-watching as a non-lethal consumptive activity. J. Sustain. Tour..

[31] Kubo T, Mieno T, Kuriyama K (2019). Wildlife viewing: The impact of money-back guarantees. Tour. Manag..

[32] Sugimura K (2000). Distribution and abundance of the Amami rabbit *Pentalagus furnessi* in the Amami and Tokuno Islands, Japan. Oryx.

[33] Yamada, F. & Smith, A.T. *Pentalagus furnessi*. The IUCN Red List of Threatened Species 2016: e.T16559A45180151. (2016).

[34] Nash JF (1950). Equilibrium points in N-person games. PNAS.

[35] Maschler, M., Solan, E. & Zamir, S. *Game Theory* (Cambridge University Press, Cambridge, 2013).

[36] Stiglitz, J. E. & Walsh, C. E. *Economics* (W.W. Norton & Company, New York, 2006), 4th edn.

[37] Nowak, M. A. *Evolutionary Dynamics: Exploring the Equations of Life* (Harvard University Press, Cambridge, 2006).

[38] Fehr E, Gächter S (2002). Altruistic punishment in humans. Nat..

[39] Fehr E, Fischbacher U (2003). The nature of human altruism. Nat..

[40] Buckels EE, Jones DN, Paulhus DL (2013). Behavioral confirmation of everyday sadism. Psychol. Sci..

[41] Fehr E, Glätzle-Rützler D, Sutter M (2013). The development of egalitarianism, altruism, spite and parochialism in childhood and adolescence. Eur. Econ. Rev..

[42] Buckels EE, Trapnell PD, Paulhus DL (2014). Trolls just want to have fun. Pers. Individ. Differ..

[43] Capraro V (2015). The emergence of hyper-altruistic behaviour in conflictual situations. Sci. Reports.

[44] Zhao K, Ferguson E, Smillie LD (2016). Prosocial personality traits differentially predict egalitarianism, generosity, and reciprocity in economic games. Front. Psychol..

[45] Judge TA, Higgins CA, Thoresen CJ, Barrick MR (1999). The Big Five personality traits, general mental ability, and career success across the life span. Pers. Psychol..

[46] Paulhus DL, Williams KM (2002). The Dark Triad of personality: Narcissism, Machiavellianism, and psychopathy. J. Res. Pers..

[47] Lee K, Ashton MC (2005). Psychopathy, Machiavellianism, and Narcissism in the Five-Factor Model and the HEXACO model of personality structure. Pers. Individ. Differ..

[48] Furnham A, Richards SC, Paulhus DL (2013). The Dark Triad of personality: A 10 year review. Soc. Pers. Psychol. Compass.

[49] Kvasova O (2015). The Big Five personality traits as antecedents of eco-friendly tourist behavior. Pers. Individ. Differ..

[50] Scatà M, DiStefano A, Liò P, LaCorte A (2016). The impact of heterogeneity and awareness in modeling epidemic spreading on multiplex networks. Sci. Reports.

[51] Capraro, V. & Halpern, J. Y. Translucent players: Explaining cooperative behavior in social dilemmas. *Proc. 15th Conf. on Theor. Aspects Ration. Knowledge, 2015* (2018).

[52] Capraro V, Rand DG (2018). Do the right thing: Experimental evidence that preferences for moral behavior, rather than equity or efficiency per se, drive human prosociality. Judgm. Decis. Mak..

[53] Benjamin C, Sarkar S (2019). Triggers for cooperative behavior in the thermodynamic limit: A case study in Public goods game. Chaos.

[54] Fehr E, Gächter S (2000). Cooperation and punishment in public goods experiments. Am. Econ. Rev..

[55] Milinski M, Semmann D, Krambeck H-J (2002). Reputation helps solve the tragedy of the commons. Nat..

[56] Nowak MA, Sigmund K (2005). Evolution of indirect reciprocity. Nat..

[57] Nowak MA (2006). Five rules for the evolution of cooperation. Sci..

[58] Capraro V, Giardini F, Vilone D, Paolucci M (2016). Partner selection supported by opaque reputation promotes cooperative behavior. Judgm. Decis. Mak..

