# Supplementary material for: Social Dilemmas in Nature-Based Tourism Depend on Social Value Orientations
Source: Sci Rep. 2020 Feb 28;10:3730. doi: 10.1038/s41598-020-60349-z (PMC7048808; doi:10.1038/s41598-020-60349-z)
Supplement: Supplementary file 1 — Supplementary Information [file 41598_2020_60349_MOESM1_ESM.pdf]

Supplementary Information: Social Dilemmas in Nature-Based Tourism Depend on Social Value Orientations

Keita Honjo Takahiro Kubo

January 20, 2020

1 Nash equilibria of the SPBM

As shown by equation (4), the SPBM of the WVG is

$$V = \begin{bmatrix} ((\alpha_1+\beta_1)U_1^{\mathrm{TT}}, (\alpha_2+\beta_2)U_1^{\mathrm{TT}}) & (\alpha_1 U_1^{\mathrm{TF}}, \beta_2 U_1^{\mathrm{TF}}) \\ (\beta_1 U_1^{\mathrm{TF}}, \alpha_2 U_1^{\mathrm{TF}}) & (0,0) \end{bmatrix}. \tag{S1}$$

Here, we identify the conditions for which the SPBM has Nash equilibria.

1.1 (F,F)-Nash

The strategy profile (F,F) is a Nash equilibrium if

$$\forall i \in \mathbf{N}(\alpha_i U_1^{\mathrm{TF}} \leq 0). \tag{S2}$$

The individualistic, competitive, and prosocial players have positive values of α_i. Hence, we can reduce the equilibrium condition to $U_1^{\mathrm{TF}} \leq 0$. Solving this inequality with respect to the initial encounter probability gives

$$P \leq P_0 = \frac{C - \delta B}{B(1-\delta)}. \tag{S3}$$

The condition for (F,F)-Nash is independent of players' SVOs.

1.2 (T,T)-Nash

The strategy profile (T,T) is a Nash equilibrium if

$$\forall i \mathrm{BR}_i^{\mathrm{TT}} \iff \forall i((\alpha_i+\beta_i)U_1^{\mathrm{TT}} \geq \beta_i U_1^{\mathrm{TF}}), \tag{S4}$$

where $\mathrm{BR}_i^{s_1 s_2}$ denotes the condition for which player i chooses the best response s_i to the other player's strategy s_{-i}. If player i is individualistic ($\alpha_i = 1$ and $\beta_i = 0$),

$$\mathrm{BR}_i^{\mathrm{TT}} \iff P \geq \frac{P_0}{\theta}. \tag{S5}$$

If player i is competitive ($\alpha_i = 1/\sqrt{2}$ and $\beta_i = -1/\sqrt{2}$),

$$\mathrm{BR}_i^{\mathrm{TT}} \iff P \geq P_0. \tag{S6}$$

If player i is prosocial ($\alpha_i = 1/\sqrt{2}$ and $\beta_i = 1/\sqrt{2}$),

$$\mathrm{BR}_i^{\mathrm{TT}} \iff \begin{cases} P \leq \frac{P_0}{2\theta-1} & (\theta < \frac{1}{2}) \\ \delta \geq \frac{C}{B} & (\theta = \frac{1}{2}) \\ P \geq \frac{P_0}{2\theta-1} & (\theta > \frac{1}{2}) \end{cases}. \tag{S7}$$

1.3 (T,F)-Nash and (F,T)-Nash

The strategy profile (T,F) is a Nash equilibrium if

$$\forall i \mathrm{BR}_i^{\mathrm{TF}} \iff (\alpha_1 U_1^{\mathrm{TF}} \geq 0) \wedge ((\alpha_2 + \beta_2) U_1^{\mathrm{TT}} \leq \beta_2 U_1^{\mathrm{TF}}). \tag{S8}$$

The first condition can be reduced to $P \geq P_0$, which is independent of players' SVOs. The equilibrium condition depends only on the SVO of player 2. If player 2 is individualistic, the condition for (T,F)-Nash is

$$\forall i \mathrm{BR}_i^{\mathrm{TF}} \iff (P \geq P_0) \wedge \left(P \leq \frac{P_0}{\theta}\right). \tag{S9}$$

If player 2 is competitive,

$$\forall i \mathrm{BR}_i^{\mathrm{TF}} \iff P = P_0. \tag{S10}$$

If player 2 is prosocial,

$$\forall i \mathrm{BR}_i^{\mathrm{TF}} \iff \begin{cases} (P \geq P_0) \wedge (P \geq \frac{P_0}{2\theta - 1}) & (\theta < \frac{1}{2}) \\ (P \geq P_0) \wedge \left(\delta \leq \frac{C}{B}\right) & (\theta = \frac{1}{2}) \\ (P \geq P_0) \wedge (P \leq \frac{P_0}{2\theta - 1}) & (\theta > \frac{1}{2}) \end{cases}. \tag{S11}$$

The condition for (F,T)-Nash is obtained in the same way.

1.4 Result

Tables S1–S3 list the conditions for Nash equilibria in the cases of $\delta < C/B$, $\delta = C/B$, and $\delta > C/B$, respectively. $G_{\tau_1\tau_2}$ denotes a WVG with player i of type $\tau_i \in \{\mathrm{I},\mathrm{C},\mathrm{P}\}$. The symbols I, P, and C are abbreviations for individualistic, competitive, and prosocial types, respectively. $\varnothing$ denotes the empty set. The proposition $P \in \varnothing$ means that the equilibrium does not exist.

Table S1: Conditions for which the SPBM has Nash equilibria ($\delta < C/B$)

Game	(F,F)-Nash	(F,T)-Nash	(T,F)-Nash	(T,T)-Nash
G_{II}	$P \in [0, P_0]$	$P \in [P_0, P_0/\theta]$	$P \in [P_0, P_0/\theta]$	$P \in [P_0/\theta, 1]$
G_{CC}	$P \in [0, P_0]$	$P = P_0$	$P = P_0$	$P \in [P_0, 1]$
G_{PP} $(\theta < 1/2)$	$P \in [0, P_0]$	$P \in [P_0, 1]$	$P \in [P_0, 1]$	$P \in \varnothing$
G_{PP} $(\theta = 1/2)$	$P \in [0, P_0]$	$P \in [P_0, 1]$	$P \in [P_0, 1]$	$P \in \varnothing$
G_{PP} $(\theta > 1/2)$	$P \in [0, P_0]$	$P \in [P_0, P_0/(2\theta - 1)]$	$P \in [P_0, P_0/(2\theta - 1)]$	$P \in [P_0/(2\theta - 1), 1]$
G_{IC}	$P \in [0, P_0]$	$P \in [P_0, P_0/\theta]$	$P = P_0$	$P \in [P_0/\theta, 1]$
G_{IP} $(\theta < 1/2)$	$P \in [0, P_0]$	$P \in [P_0, P_0/\theta]$	$P \in [P_0, 1]$	$P \in \varnothing$
G_{IP} $(\theta = 1/2)$	$P \in [0, P_0]$	$P \in [P_0, P_0/\theta]$	$P \in [P_0, 1]$	$P \in \varnothing$
G_{IP} $(\theta > 1/2)$	$P \in [0, P_0]$	$P \in [P_0, P_0/\theta]$	$P \in [P_0, P_0/(2\theta - 1)]$	$P \in [P_0/(2\theta - 1), 1]$
G_{CP} $(\theta < 1/2)$	$P \in [0, P_0]$	$P = P_0$	$P \in [P_0, 1]$	$P \in \varnothing$
G_{CP} $(\theta = 1/2)$	$P \in [0, P_0]$	$P = P_0$	$P \in [P_0, 1]$	$P \in \varnothing$
G_{CP} $(\theta > 1/2)$	$P \in [0, P_0]$	$P = P_0$	$P \in [P_0, P_0/(2\theta - 1)]$	$P \in [P_0/(2\theta - 1), 1]$

Table S2: Conditions for which the SPBM has Nash equilibria ($\delta = C/B$)

Game	(F,F)-Nash	(F,T)-Nash	(T,F)-Nash	(T,T)-Nash
G_{II}	$P = 0$	$P = 0$	$P = 0$	$P \in [0, 1]$
G_{CC}	$P = 0$	$P = 0$	$P = 0$	$P \in [0, 1]$
G_{PP} $(\theta < 1/2)$	$P = 0$	$P \in [0, 1]$	$P \in [0, 1]$	$P = 0$
G_{PP} $(\theta = 1/2)$	$P = 0$	$P \in [0, 1]$	$P \in [0, 1]$	$P \in [0, 1]$
G_{PP} $(\theta > 1/2)$	$P = 0$	$P = 0$	$P = 0$	$P \in [0, 1]$
G_{IC}	$P = 0$	$P = 0$	$P = 0$	$P \in [0, 1]$
G_{IP} $(\theta < 1/2)$	$P = 0$	$P = 0$	$P \in [0, 1]$	$P = 0$
G_{IP} $(\theta = 1/2)$	$P = 0$	$P = 0$	$P \in [0, 1]$	$P \in [0, 1]$
G_{IP} $(\theta > 1/2)$	$P = 0$	$P = 0$	$P = 0$	$P \in [0, 1]$
G_{CP} $(\theta < 1/2)$	$P = 0$	$P = 0$	$P \in [0, 1]$	$P = 0$
G_{CP} $(\theta = 1/2)$	$P = 0$	$P = 0$	$P \in [0, 1]$	$P \in [0, 1]$
G_{CP} $(\theta > 1/2)$	$P = 0$	$P = 0$	$P = 0$	$P \in [0, 1]$

Table S3: Conditions for which the SPBM has Nash equilibria ($\delta > C/B$)

Game	(F,F)-Nash	(F,T)-Nash	(T,F)-Nash	(T,T)-Nash
G_{II}	$P \in \varnothing$	$P \in \varnothing$	$P \in \varnothing$	$P \in [0, 1]$
G_{CC}	$P \in \varnothing$	$P \in \varnothing$	$P \in \varnothing$	$P \in [0, 1]$
G_{PP} $(\theta < 1/2)$	$P \in \varnothing$	$P \in [P_0/(2\theta - 1), 1]$	$P \in [P_0/(2\theta - 1), 1]$	$P \in [0, P_0/(2\theta - 1)]$
G_{PP} $(\theta = 1/2)$	$P \in \varnothing$	$P \in \varnothing$	$P \in \varnothing$	$P \in [0, 1]$
G_{PP} $(\theta > 1/2)$	$P \in \varnothing$	$P \in \varnothing$	$P \in \varnothing$	$P \in [0, 1]$
G_{IC}	$P \in \varnothing$	$P \in \varnothing$	$P \in \varnothing$	$P \in [0, 1]$
G_{IP} $(\theta < 1/2)$	$P \in \varnothing$	$P \in \varnothing$	$P \in [P_0/(2\theta - 1), 1]$	$P \in [0, P_0/(2\theta - 1)]$
G_{IP} $(\theta = 1/2)$	$P \in \varnothing$	$P \in \varnothing$	$P \in \varnothing$	$P \in [0, 1]$
G_{IP} $(\theta > 1/2)$	$P \in \varnothing$	$P \in \varnothing$	$P \in \varnothing$	$P \in [0, 1]$
G_{CP} $(\theta < 1/2)$	$P \in \varnothing$	$P \in \varnothing$	$P \in [P_0/(2\theta - 1), 1]$	$P \in [0, P_0/(2\theta - 1)]$
G_{CP} $(\theta = 1/2)$	$P \in \varnothing$	$P \in \varnothing$	$P \in \varnothing$	$P \in [0, 1]$
G_{CP} $(\theta > 1/2)$	$P \in \varnothing$	$P \in \varnothing$	$P \in \varnothing$	$P \in [0, 1]$

2 Pareto-efficient solutions of the OPBM

As shown by equation (2), the OPBM of the WVG is

$$U = \begin{bmatrix} (U_1^{\mathrm{TT}}, U_1^{\mathrm{TT}}) & (U_1^{\mathrm{TF}}, 0) \\ (0, U_1^{\mathrm{TF}}) & (0, 0) \end{bmatrix}. \tag{S12}$$

Here, we identify the conditions for which the OPBM has Pareto-efficient solutions.

2.1 (F,F)-Pareto

The strategy profile (F,F) is not a Pareto-efficient solution if

$$\begin{aligned} &(u^{\mathrm{FF}} \in \mathbf{P}(u^{\mathrm{FF}})) \vee (u^{\mathrm{FT}} \in \mathbf{P}(u^{\mathrm{FF}})) \vee (u^{\mathrm{TF}} \in \mathbf{P}(u^{\mathrm{FF}})) \vee (u^{\mathrm{TT}} \in \mathbf{P}(u^{\mathrm{FF}})) \\ \iff\ & (0 < U_1^{\mathrm{TF}}) \vee (0 < U_1^{\mathrm{TT}}) \end{aligned} \tag{S13}$$

The condition for (F,F)-Pareto is

$$(0 \geq U_1^{\mathrm{TF}}) \wedge (0 \geq U_1^{\mathrm{TT}}) \iff (P \leq P_0) \wedge \left(P \leq \frac{P_0}{\theta}\right). \tag{S14}$$

2.2 (T,T)-Pareto

The strategy profile (T,T) is not a Pareto-efficient solution if

$$\begin{aligned}
&(u^{\mathrm{FF}} \in \mathbf{P}(u^{\mathrm{TT}})) \vee (u^{\mathrm{FT}} \in \mathbf{P}(u^{\mathrm{TT}})) \vee (u^{\mathrm{TF}} \in \mathbf{P}(u^{\mathrm{TT}})) \vee (u^{\mathrm{TT}} \in \mathbf{P}(u^{\mathrm{TT}}))\\
\iff& ((U_1^{\mathrm{TT}} = U_1^{\mathrm{TF}}) \wedge (U_1^{\mathrm{TT}} < 0)) \vee ((U_1^{\mathrm{TT}} < U_1^{\mathrm{TF}}) \wedge (U_1^{\mathrm{TT}} \leq 0)) \vee (U_1^{\mathrm{TT}} < 0)\\
\iff& (U_1^{\mathrm{TT}} < 0) \vee ((U_1^{\mathrm{TT}} < U_1^{\mathrm{TF}}) \wedge (U_1^{\mathrm{TT}} \leq 0))
\end{aligned} \tag{S15}$$

The condition for (T,T)-Pareto is

$$\begin{aligned}
&(U_1^{\mathrm{TT}} \geq 0) \wedge ((U_1^{\mathrm{TT}} \geq U_1^{\mathrm{TF}}) \vee (U_1^{\mathrm{TT}} > 0))\\
\iff& \left(P \geq \frac{P_0}{\theta}\right) \wedge \left((P = 0) \vee \left(P > \frac{P_0}{\theta}\right)\right).
\end{aligned} \tag{S16}$$

2.3 (T,F)-Pareto and (F,T)-Pareto

The strategy profile (T,F) is not a Pareto-efficient solution if

$$\begin{aligned}
&(u^{\mathrm{FF}} \in \mathbf{P}(u^{\mathrm{TF}})) \vee (u^{\mathrm{FT}} \in \mathbf{P}(u^{\mathrm{TF}})) \vee (u^{\mathrm{TF}} \in \mathbf{P}(u^{\mathrm{TF}})) \vee (u^{\mathrm{TT}} \in \mathbf{P}(u^{\mathrm{TF}}))\\
\iff& (U_1^{\mathrm{TF}} < 0) \vee ((U_1^{\mathrm{TF}} = U_1^{\mathrm{TT}}) \wedge (0 < U_1^{\mathrm{TT}})).
\end{aligned} \tag{S17}$$

The condition for (T,F)-Pareto is

$$\begin{aligned}
&(U_1^{\mathrm{TF}} \geq 0) \wedge ((U_1^{\mathrm{TF}} \neq U_1^{\mathrm{TT}}) \vee (0 \geq U_1^{\mathrm{TF}}))\\
\iff& (P \geq P_0) \wedge \left((P > 0) \vee \left(P \leq \frac{P_0}{\theta}\right)\right).
\end{aligned} \tag{S18}$$

As the OPBM is symmetric, (F,T)-Pareto always coexists with (T,F)-Pareto.

2.4 Result

Table S4 lists the conditions for which the OPBM has Pareto-efficient solutions. As the individual, competitive, and prosocial players have the same OPBM, the solution conditions are independent of players' SVOs.

Table S4: Conditions for which the OPBM has Pareto-efficient solutions

Guarantee coef.	(F,F)-Pareto	(F,T)-Pareto	(T,F)-Pareto	(T,T)-Pareto
$\delta < C/B$	$P \in [0, P_0]$	$P \in [P_0, 1]$	$P \in [P_0, 1]$	$P \in (P_0/\theta, 1]$
$\delta = C/B$	$P = 0$	$P \in [0, 1]$	$P \in [0, 1]$	$P \in [0, 1]$
$\delta > C/B$	$P \in \varnothing$	$P \in (0, 1]$	$P \in (0, 1]$	$P \in [0, 1]$

3 Conditions for PINE

From the conditions for Nash equilibria and Pareto-efficient solutions (Tables S1–S4), we obtain the conditions for PINE. Tables S5–S6 show the results in the cases of $\delta < C/B$ and $\delta \geq C/B$, respectively.

Table S5: Conditions for PINE ($\delta < C/B$)

Game	(F, F)-PINE	(F, T)-PINE	(T, F)-PINE	(T, T)-PINE
G_{II}	$P \in \varnothing$	$P \in \varnothing$	$P \in \varnothing$	$P = P_0/\theta$
G_{CC}	$P \in \varnothing$	$P \in \varnothing$	$P \in \varnothing$	$P \in [P_0, P_0/\theta]$
G_{PP} ($\theta < 1/2$)	$P \in \varnothing$	$P \in \varnothing$	$P \in \varnothing$	$P \in \varnothing$
G_{PP} ($\theta = 1/2$)	$P \in \varnothing$	$P \in \varnothing$	$P \in \varnothing$	$P \in \varnothing$
G_{PP} ($\theta > 1/2$)	$P \in \varnothing$	$P \in \varnothing$	$P \in \varnothing$	$P \in \varnothing$
G_{IC}	$P \in \varnothing$	$P \in \varnothing$	$P \in \varnothing$	$P = P_0/\theta$
G_{IP} ($\theta < 1/2$)	$P \in \varnothing$	$P \in \varnothing$	$P \in \varnothing$	$P \in \varnothing$
G_{IP} ($\theta = 1/2$)	$P \in \varnothing$	$P \in \varnothing$	$P \in \varnothing$	$P \in \varnothing$
G_{IP} ($\theta > 1/2$)	$P \in \varnothing$	$P \in \varnothing$	$P \in \varnothing$	$P \in \varnothing$
G_{CP} ($\theta < 1/2$)	$P \in \varnothing$	$P \in \varnothing$	$P \in \varnothing$	$P \in \varnothing$
G_{CP} ($\theta = 1/2$)	$P \in \varnothing$	$P \in \varnothing$	$P \in \varnothing$	$P \in \varnothing$
G_{CP} ($\theta > 1/2$)	$P \in \varnothing$	$P \in \varnothing$	$P \in \varnothing$	$P \in \varnothing$

Table S6: Conditions for PINE ($\delta \geq C/B$)

Game	(F, F)-PINE	(F, T)-PINE	(T, F)-PINE	(T, T)-PINE
G_{II}	$P \in \varnothing$	$P \in \varnothing$	$P \in \varnothing$	$P \in \varnothing$
G_{CC}	$P \in \varnothing$	$P \in \varnothing$	$P \in \varnothing$	$P \in \varnothing$
G_{PP} ($\theta < 1/2$)	$P \in \varnothing$	$P \in \varnothing$	$P \in \varnothing$	$P \in \varnothing$
G_{PP} ($\theta = 1/2$)	$P \in \varnothing$	$P \in \varnothing$	$P \in \varnothing$	$P \in \varnothing$
G_{PP} ($\theta > 1/2$)	$P \in \varnothing$	$P \in \varnothing$	$P \in \varnothing$	$P \in \varnothing$
G_{IC}	$P \in \varnothing$	$P \in \varnothing$	$P \in \varnothing$	$P \in \varnothing$
G_{IP} ($\theta < 1/2$)	$P \in \varnothing$	$P \in \varnothing$	$P \in \varnothing$	$P \in \varnothing$
G_{IP} ($\theta = 1/2$)	$P \in \varnothing$	$P \in \varnothing$	$P \in \varnothing$	$P \in \varnothing$
G_{IP} ($\theta > 1/2$)	$P \in \varnothing$	$P \in \varnothing$	$P \in \varnothing$	$P \in \varnothing$
G_{CP} ($\theta < 1/2$)	$P \in \varnothing$	$P \in \varnothing$	$P \in \varnothing$	$P \in \varnothing$
G_{CP} ($\theta = 1/2$)	$P \in \varnothing$	$P \in \varnothing$	$P \in \varnothing$	$P \in \varnothing$
G_{CP} ($\theta > 1/2$)	$P \in \varnothing$	$P \in \varnothing$	$P \in \varnothing$	$P \in \varnothing$

4 Punishment effect on Nash equilibria

Here, we impose punishment on the concentration of tours and examine its effect on the emergence of Nash equilibria. As shown by equation (11), the SPBM with punishment is

$$V_\epsilon = \begin{bmatrix} ((\alpha_1 + \beta_1)(U_1^{\mathrm{TT}} - \epsilon), (\alpha_2 + \beta_2)(U_1^{\mathrm{TT}} - \epsilon)) & (\alpha_1 U_1^{\mathrm{TF}}, \beta_2 U_1^{\mathrm{TF}}) \\ (\beta_1 U_1^{\mathrm{TF}}, \alpha_2 U_1^{\mathrm{TF}}) & (0, 0) \end{bmatrix}. \tag{S19}$$

4.1 (F,F)-Nash

The punishment does not change the conditions for (F,F)-Nash.

4.2 (T,T)-Nash

The SPBM has (T,T)-Nash if

$$\forall i \mathrm{BR}_i^{\mathrm{TT}} \iff \forall i((\alpha_i+\beta_i)(U_1^{\mathrm{TT}}-\epsilon) \geq \beta_i U_1^{\mathrm{TF}}). \tag{S20}$$

When player i is individualistic,

$$\mathrm{BR}_i^{\mathrm{TT}} \iff P \geq \frac{C-\delta B+\epsilon}{\theta B(1-\delta)} = \frac{P_0+\epsilon_0}{\theta}, \tag{S21}$$

where $\epsilon_0 = \epsilon/(B(1-\delta))$. Since $\epsilon_0 > 0$, we have

$$\frac{P_0+\epsilon_0}{\theta} > \frac{P_0}{\theta}. \tag{S22}$$

When player i is competitive,

$$\mathrm{BR}_i^{\mathrm{TT}} \iff P \geq P_0, \tag{S23}$$

which is equal to the best response condition in the original game without punishment (equation (S6)). When player i is prosocial,

$$\mathrm{BR}_i^{\mathrm{TT}} \iff \begin{cases} P \leq \frac{P_0+2\epsilon_0}{2\theta-1} & (\theta < \frac{1}{2}) \\ \delta \geq \frac{C+2\epsilon}{B} & (\theta = \frac{1}{2}) \\ P \geq \frac{P_0+2\epsilon_0}{2\theta-1} & (\theta > \frac{1}{2}) \end{cases}. \tag{S24}$$

Since $\epsilon > 0$,

$$\frac{C+2\epsilon}{B} > \frac{C}{B}. \tag{S25}$$

4.3 (T,F)-Nash and (F,T)-Nash

The SPBM has (T,F)-Nash if

$$\forall i \mathrm{BR}_i^{\mathrm{TF}} \iff (\alpha_1 U_1^{\mathrm{TF}} \geq 0) \wedge ((\alpha_2+\beta_2)(U_1^{\mathrm{TT}}-\epsilon) \leq \beta_2 U_1^{\mathrm{TF}}). \tag{S26}$$

The equilibrium condition depends only on the SVO of player 2. If player 2 is individualistic,

$$\forall i \mathrm{BR}_i^{\mathrm{TF}} \iff (P \geq P_0) \wedge \left(P \leq \frac{P_0+\epsilon_0}{\theta}\right). \tag{S27}$$

If player 2 is competitive,

$$\forall i \mathrm{BR}_i^{\mathrm{TF}} \iff P = P_0. \tag{S28}$$

If player 2 is prosocial,

$$\forall i \mathrm{BR}_i^{\mathrm{TF}} \iff \begin{cases} (P \geq P_0) \wedge (P \geq \frac{P_0+2\epsilon_0}{2\theta-1}) & (\theta < \frac{1}{2}) \\ (P \geq P_0) \wedge (\delta \leq \frac{C+2\epsilon}{B}) & (\theta = \frac{1}{2}) \\ (P \geq P_0) \wedge (P \leq \frac{P_0+2\epsilon_0}{2\theta-1}) & (\theta > \frac{1}{2}) \end{cases}. \tag{S29}$$

The condition for (F,T)-Nash is obtained in the same way.

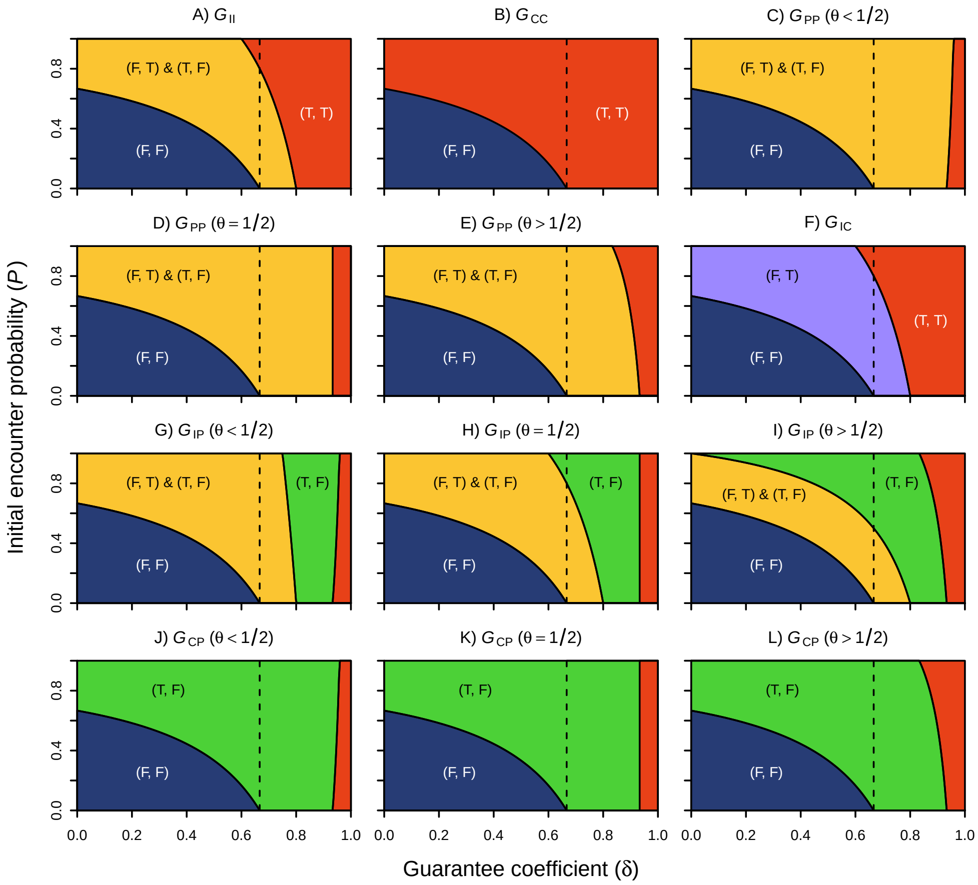

Figure S1: Nash equilibria of the SPBM with punishment on the strategy profile (T, T) ($B = 1.5$, $C = 1.0$, $\theta \in \{0.2, 0.5, 0.8\}$, and $\epsilon = 0.2$). Panels C, G, and J assume $\theta = 0.2$. Panels E, I, and L assume $\theta = 0.8$. The other panels assume $\theta = 0.5$. $G_{\tau_1 \tau_2}$ denotes a WVG with player i of type $\tau_i \in \{\mathrm{I}, \mathrm{C}, \mathrm{P}\}$. The symbols I, C, and P are abbreviations for individualistic, competitive, and prosocial types, respectively. The dashed line indicates $\delta = C/B$.

4.4 Result

Figure S1 shows the distribution of Nash equilibria over the parameter space $\mathbf{D} = \{(\delta, P) \mid \delta \in (0, 1), P \in [0, 1]\}$. The parameter values are the same as Figure 3 except for ϵ. The punishment does not influence the equilibrium distribution of G_{CC}, while it decreases the region of (T, T)-Nash in all the other games.

5 Punishment effect on Pareto-efficient solutions

Here, we examine the effect of fair punishment on the emergence of Pareto-efficient solutions. As shown by equation (10), the OPBM with punishment is

$$U_\epsilon = \begin{bmatrix} (U_1^{\mathrm{TT}} - \epsilon, U_1^{\mathrm{TT}} - \epsilon) & (U_1^{\mathrm{TF}}, 0) \\ (0, U_1^{\mathrm{TF}}) & (0, 0) \end{bmatrix}. \tag{S30}$$

5.1 (F,F)-Pareto

The strategy profile (F,F) is not a Pareto-efficient solution if

$$\begin{aligned}&(u^{\mathrm{FF}} \in \mathbf{P}(u^{\mathrm{FF}})) \vee (u^{\mathrm{FT}} \in \mathbf{P}(u^{\mathrm{FF}})) \vee (u^{\mathrm{TF}} \in \mathbf{P}(u^{\mathrm{FF}})) \vee (u^{\mathrm{TT}} \in \mathbf{P}(u^{\mathrm{FF}}))\\ \iff &(0 < U_1^{\mathrm{TF}}) \vee (0 < U_1^{\mathrm{TT}} - \epsilon).\end{aligned} \tag{S31}$$

The condition for (F,F)-Pareto is

$$(0 \geq U_1^{\mathrm{TF}}) \wedge (0 \geq U_1^{\mathrm{TT}} - \epsilon) \iff (P \leq P_0) \wedge \left(P \leq \frac{P_0 + \epsilon_0}{\theta}\right). \tag{S32}$$

5.2 (T,T)-Pareto

The strategy profile (T,T) is not a Pareto-efficient solution if

$$\begin{aligned}&(u^{\mathrm{FF}} \in \mathbf{P}(u^{\mathrm{TT}})) \vee (u^{\mathrm{FT}} \in \mathbf{P}(u^{\mathrm{TT}})) \vee (u^{\mathrm{TF}} \in \mathbf{P}(u^{\mathrm{TT}})) \vee (u^{\mathrm{TT}} \in \mathbf{P}(u^{\mathrm{TT}}))\\ \iff &U_1^{\mathrm{TT}} - \epsilon \leq 0\end{aligned} \tag{S33}$$

The condition for (T,T)-Pareto is

$$U_1^{\mathrm{TT}} - \epsilon > 0 \iff P > \frac{P_0 + \epsilon_0}{\theta}. \tag{S34}$$

5.3 (T,F)-Pareto and (F,T)-Pareto

The strategy profile (T,F) is not a Pareto-efficient solution if

$$\begin{aligned}&(u^{\mathrm{FF}} \in \mathbf{P}(u^{\mathrm{TF}})) \vee (u^{\mathrm{FT}} \in \mathbf{P}(u^{\mathrm{TF}})) \vee (u^{\mathrm{TF}} \in \mathbf{P}(u^{\mathrm{TF}})) \vee (u^{\mathrm{TT}} \in \mathbf{P}(u^{\mathrm{TF}}))\\ \iff &U_1^{\mathrm{TF}} < 0.\end{aligned} \tag{S35}$$

The condition for (T,F)-Pareto is

$$U_1^{\mathrm{TF}} \geq 0 \iff P \geq P_0. \tag{S36}$$

As the OPBM is symmetric, (F,T)-Pareto always coexists with (T,F)-Pareto.

5.4 Result

Figure S2 shows the distribution of Pareto-efficient solutions over the parameter space $\mathbf{D}$. The parameter values are the same as Figure 4 except for ϵ. The region of (T,T)-Pareto is narrower than in the game without punishment.

6 Examples of SVO-based non-cooperative games

Here, we apply the SVO-based approach to three non-cooperative games: the Hawk-Dove game, Battle of the Sexes, and Public Goods game (Nowak, 2006; Maschler et al., 2013). For simplicity, we assume that two players have the same SVO.

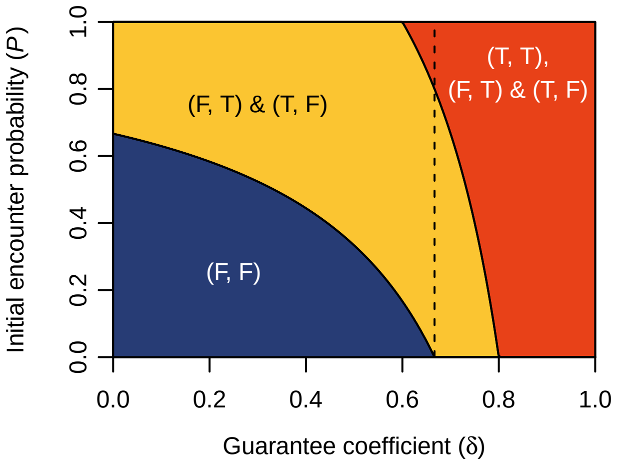

Figure S2: Pareto-efficient solutions of the OPBM with punishment on the strategy profile (T, T) ($B = 1.5$, $C = 1.0$, $\theta = 0.5$, and $\epsilon = 0.2$). The dashed line indicates $\delta = C/B$.

6.1 Hawk-Dove game

The OPBM of the Hawk-Dove game is expressed as

$$\begin{bmatrix} (\mathrm{H}, \mathrm{H}) & (\mathrm{H}, \mathrm{D}) \\ (\mathrm{D}, \mathrm{H}) & (\mathrm{D}, \mathrm{D}) \end{bmatrix} = \begin{bmatrix} ((b-c)/2, (b-c)/2) & (b, 0) \\ (0, b) & (b/2, b/2) \end{bmatrix}, \tag{S37}$$

where strategies H and D indicate Hawk and Dove, respectively. It is assumed that $0 < b < c$.

If players are individualistic, the SPBM of the game is equal to the OPBM. The SPBM has two Nash equilibria (D, H) and (H, D). The objective payoff allocations given by the solutions are Pareto efficient.

If players are prosocial, the SPBM of the game is

$$\begin{bmatrix} ((b-c)/\sqrt{2}, (b-c)/\sqrt{2}) & (b/\sqrt{2}, b/\sqrt{2}) \\ (b/\sqrt{2}, b/\sqrt{2}) & (b/\sqrt{2}, b/\sqrt{2}) \end{bmatrix}, \tag{S38}$$

which has three Nash equilibria (D, H), (H, D), and (D, D). The objective payoff allocations given by the solutions are Pareto efficient.

If players are competitive, the SPBM of the game is

$$\begin{bmatrix} (0, 0) & (b/\sqrt{2}, -b/\sqrt{2}) \\ (-b/\sqrt{2}, b/\sqrt{2}) & (0, 0) \end{bmatrix}. \tag{S39}$$

In this case, H is the dominant strategy for both players, and the SPBM has the unique Nash equilibrium (H, H). The objective payoff allocation given by the solution is not Pareto efficient. By moving from (H, H) to (D, D), each of the players can improve his or her objective payoff.

In the Hawk-Dove game, individualistic and prosocial players achieve Pareto-efficient Nash equilibria. In contrast, competitive players are attracted to a PINE. Similar to the WVG, the Hawk-Dove game is a potential social dilemma.

6.2 Battle of the Sexes

The OPBM of the Battle of the Sexes is

$$\begin{bmatrix} (\mathrm{F}, \mathrm{F}) & (\mathrm{F}, \mathrm{C}) \\ (\mathrm{C}, \mathrm{F}) & (\mathrm{C}, \mathrm{C}) \end{bmatrix} = \begin{bmatrix} (2, 1) & (0, 0) \\ (0, 0) & (1, 2) \end{bmatrix}, \tag{S40}$$

where strategies F and C indicate Football and Concert, respectively.

If players are individualistic, the SPBM of the game is equal to the OPBM. The SPBM has two Nash equilibria (F, F) and (C, C). The objective payoff allocations given by the solutions are Pareto efficient.

If players are prosocial, the SPBM of the game is

$$\begin{bmatrix} (3/\sqrt{2}, 3/\sqrt{2}) & (0,0) \\ (0,0) & (3/\sqrt{2}, 3/\sqrt{2}) \end{bmatrix}, \tag{S41}$$

which has the same solutions as the game played by individualistic players.

If players are competitive, the SPBM of the game is

$$\begin{bmatrix} (1/\sqrt{2}, -1/\sqrt{2}) & (0,0) \\ (0,0) & (-1/\sqrt{2}, 1/\sqrt{2}) \end{bmatrix}. \tag{S42}$$

In this case, F is the dominant strategy for player 1, while C is the dominant strategy for player 2. Therefore, (F, C) is the unique Nash equilibrium, which gives a Pareto-inefficient objective payoff allocation.

In the Battle of the Sexes, individualistic and prosocial players achieve Pareto-efficient Nash equilibria, but competitive players are attracted to a PINE. Similar to the WVG, the Battle of the Sexes is a potential social dilemma.

6.3 Public Goods game

Assume that each player $i \in \{1, 2\}$ has an initial endowment $c_i > 0$ and provides public goods by paying $x_i \in [0, c_i]$. Player i's objective payoff is given by $c_i - x_i + \alpha(x_1 + x_2)$. α is a parameter satisfying $1/2 < \alpha < 1$. The OPBM of the Public Goods game is expressed as

$$\begin{bmatrix} (\mathrm{C},\mathrm{C}) & (\mathrm{C},\mathrm{D}) \\ (\mathrm{D},\mathrm{C}) & (\mathrm{D},\mathrm{D}) \end{bmatrix} = \begin{bmatrix} (\alpha(x_1+x_2), \alpha(x_1+x_2)) & (\alpha x_1, \alpha x_1 + x_2) \\ (x_1 + \alpha x_2, \alpha x_2) & (x_1, x_2) \end{bmatrix}, \tag{S43}$$

where strategies C and D indicate Cooperate and Defect, respectively. A player with strategy C contributes to public goods ($x_i > 0$), while a player with strategy D is a free rider ($x_i = 0$). In this game, the strategy profile (C, C) is the solution that maximises the total objective payoff $U_1 + U_2$.

If players are individualistic, the SPBM of the game is equal to the OPBM. D is the dominant strategy for both players, and the SPBM has the unique Nash equilibrium (D, D).

If players are competitive, the SPBM of the game is

$$\begin{bmatrix} (0,0) & (-x_2/\sqrt{2}, x_2/\sqrt{2}) \\ (x_1/\sqrt{2}, -x_1/\sqrt{2}) & ((x_1-x_2)/\sqrt{2}, (x_2-x_1)/\sqrt{2}) \end{bmatrix}, \tag{S44}$$

where D is the dominant strategy for both players. Similar to individualistic players, (D, D) is the unique Nash equilibrium.

If players are prosocial, the SPBM of the game is

$$\begin{bmatrix} (\sqrt{2}\alpha(x_1+x_2), \sqrt{2}\alpha(x_1+x_2)) & ((2\alpha x_1 + x_2)/\sqrt{2}, (2\alpha x_1 + x_2)/\sqrt{2}) \\ ((x_1 + 2\alpha x_2)/\sqrt{2}, (x_1 + 2\alpha x_2)/\sqrt{2}) & ((x_1+x_2)/\sqrt{2}, (x_1+x_2)/\sqrt{2}) \end{bmatrix}. \tag{S45}$$

In this case, C is the dominant strategy for both players, and the SPBM has the unique Nash equilibrium (C, C).

In a Public Goods game with a sufficiently high α, the total objective payoff is maximised when both players are prosocial.

7 Behaviours of altruistic and sadistic players in the WVG

As shown by Figure 1, altruistic and sadistic players are characterised by the weight vectors $(\alpha_i, \beta_i) = (0, 1)$ and $(\alpha_i, \beta_i) = (0, -1)$, respectively. Both of the players have no interest in their own objective payoffs and make decisions based on the other's objective payoff. With an increase in the other's objective payoff, the subjective payoff increases for the altruistic player but decreases for the sadistic player.

When players are altruistic, the SPBM of the WVG is

$$V = \begin{bmatrix} (U_1^{\mathrm{TT}}, U_1^{\mathrm{TT}}) & (0, U_1^{\mathrm{TF}}) \\ (U_1^{\mathrm{TF}}, 0) & (0, 0) \end{bmatrix}. \tag{S46}$$

If $P > 0$, the SPBM has three Nash equilibria: (T, F), (F, T), and (F, F). The concentration of tours, denoted by (T, T), does not emerge in the altruistic market. If $P = 0$, U_1^{TT} is equal to U_1^{TF}, and all the strategy profiles are Nash equilibria. The strategy choice of the altruistic player is independent of the game parameters. In other words, the Nash equilibria hold at any point of the parameter region $\mathbf{D}$. From Figure 4, we immediately find that the objective payoff allocations given by the Nash equilibria are not always Pareto efficient.

When players are sadistic, the SPBM of the WVG is

$$V = \begin{bmatrix} (-U_1^{\mathrm{TT}}, -U_1^{\mathrm{TT}}) & (0, -U_1^{\mathrm{TF}}) \\ (-U_1^{\mathrm{TF}}, 0) & (0, 0) \end{bmatrix}. \tag{S47}$$

If $P > 0$, the SPBM has two Nash equilibria (T, T) and (F, F). If $P = 0$, all the strategy profiles are Nash equilibria. Similar to the altruistic player, the strategy choice of the sadistic player is also independent of the game parameters. The objective payoff allocations given by the Nash equilibria are not always Pareto efficient.
